# Incidence of idiopathic intracranial hypertension (IIH) in adults and children: a 14-year population-based study

**DOI:** 10.1007/s00415-026-13901-2

**Published:** 2026-06-13

**Authors:** Mina Botrous, Sujan Surendran, Alex Sarossy, Blake D. Colman, Paul G. Sanfillippo, Yong Lin Wang, Shivanand Sheth, Subahari Raviskanthan, David Darby, Clare L. Fraser, Marc Sarossy, Steve Simpson-Yap, Anneke van der Walt

**Affiliations:** 1 School of Translational Medicine, Monash University, Melbourne, Australia; 2 Department of Neurology, Alfred Health, Melbourne, Australia; 3 Department of Ophthalmology, Royal Children’s Hospital, Melbourne, Australia; 4 The Florey Institute of Neuroscience and Mental Health, The University of Melbourne, Parkville, Australia; 5 School of Population and Global Health, Neuroepidemiology Unit, The University of Melbourne, MelbourneCarlton, Australia; 6 MS Research Flagship, Menzies Institute of Medical Research, University of Tasmania, Hobart, Australia; 7 Department of Ophthalmology, Royal Victorian Eye and Ear Hospital, East Melbourne, Australia; 8 Save Sight Institute, Faculty of Medicine and Health, The University of Sydney, Sydney, Australia

**Keywords:** Idiopathic intracranial hypertension, Raised intracranial pressure, Epidemiology, Children, Incidence rates Australia

## Abstract

**Background:**

Idiopathic intracranial hypertension (IIH) is characterised by raised intracranial pressure in the absence of structural pathology and with normal cerebrospinal fluid composition. IIH is strongly associated with obesity and rising incidence has been reported in northern hemisphere countries. However, paediatric and Southern Hemisphere populations, including Australia, are under-represented in the existing literature.

**Methods:**

We conducted a multicentre retrospective study of adults (≥ 18 years) and children (< 18 years) diagnosed with definite IIH (Revised Friedman criteria 2013) between 2010 and 2023 at two Melbourne-based quaternary hospitals, capturing the majority of cases in Victoria, Australia. Demographic and clinical data were collected at diagnosis. Annual crude and age-standardised incidence rates per 100,000 population were calculated overall and by age group across predefined periods (2010–2014, 2015–2019, and 2020–2023). Temporal trends were assessed using negative-binomial regression.

**Results:**

A total of 612 incident IIH cases were identified, including 504 adults and 108 children. Adults were predominantly female (94%), with mean age 28.0 ± 6.5 years and mean BMI 37.6 ± 8.4 kg/m2. Children showed lower female predominance (76%), younger mean age (12.4 ± 3.5 years), and lower mean BMI (31.9 ± 8.4 kg/m2). Headache was the most common presenting symptom in both groups (adults 84% and children 86%). Diplopia and sixth nerve palsy were more frequent in children.

Age-standardised IIH incidence increased from 0.86/100,000 person-years in 2010–2014 to 6.53/100,000 in 2020–2023. In adults, incidence rose from 0.07/100,000 in 2010 to 2.30/100,000 in 2023 (32-fold); in children, from 0.33/100,000 to 1.01/100,000 (threefold). The highest incidence rates were seen among females aged 18–39 years, in whom incidence rose from 0.11/100,000 to 6.79/100,000 between 2010 and 2023.

**Conclusion:**

IIH incidence in Victoria increased substantially over 14 years, with the greatest burden among young adult females, mirroring international trends and highlighting the need for ongoing surveillance and mechanistic research.

**Supplementary Information:**

The online version contains supplementary material available at 10.1007/s00415-026-13901-2.

## Introduction

Idiopathic intracranial hypertension (IIH) is a disease that is defined by raised intracranial pressure (ICP) in the absence of an identifiable structural aetiology and normal cerebrospinal fluid composition [1, 2]. IIH typically affects obese females in their childbearing years, with peak rates of diagnosis among those aged 20–40 years, reaching approximately 3.5/100,000 in females aged 15–44 years [3, 4]. Although much rarer, IIH is well known to occur in children, with most cases reported after the onset of puberty [5]. The condition carries substantial healthcare and socioeconomic burdens, with costs reported as $444 million/ year in 2007 in the US to £49.9 million in the UK in 2014 [6, 7].

Symptoms reflect the increase in intracranial pressure and include headaches, visual disturbances, pulsatile tinnitus, and cognitive dysfunction [8, 9]. Cognitive testing demonstrates impairment in sustained attention and executive function in IIH patients compared with BMI-matched healthy controls. However, recent longitudinal studies have shown that these cognitive deficits are reversible, in line with decreased intracranial pressure [10]. There is a small but significant risk of serious permanent visual loss [11]. In cases of progressive or vision-threatening disease, surgical intervention may be required. Surgical treatment achieves high rates of papilloedema resolution and overall visual stabilisation across optic nerve sheath fenestration (ONSF), cerebrospinal fluid (CSF) diversion, and venous sinus stenting (VSS). Visual acuity improves modestly overall (− 0.13 logMAR), with the largest gains in ONSF patients reflecting worse baseline vision, and no significant difference between modalities [12].

IIH diagnosis is made based on the Revised Friedman criteria, which rely on clinical features, particularly papilloedema, and supportive neuro-imaging features [13]. Higher body mass index (BMI) at diagnosis is associated with worse visual outcomes; specifically, each one-unit increase in BMI is associated with a 0.075 dB reduction in mean deviation (MD) on visual field testing [14]. Structural measures of disease severity also predict visual prognosis. Patients with severe papilloedema (retinal nerve fibre layer [RNFL] thickness > 400 µm) showed the greatest subsequent loss of macular ganglion cell layer (GCL) volume. This decline is evident more than 12 months after baseline on visual field and optical coherence tomography (OCT) measurements. Increase in BMI and longer disease duration exert the greatest influence on visual prognosis [15].

Incidence rates of IIH have increased. In a 2014 meta-analysis of IIH incidence studies by Chen and Wall, IIH incidence rates increased over time between 1984 and 2011. This ranged from 0.03/100,000 in Japan to 0.9–2.2/100,000 in the USA and Libya in the early 1990s, and approximately 1.6/100,000 in the UK by 2011 [16]. More contemporary epidemiology studies from Wales, United Kingdom found an increase in incidence from 2.3/100,000/year in 2003 to 7.8/100,000/year in 2017 [17]. Incidence rates clearly varied between countries. In Australia, aside from a 2-year study of IIH incidence in southern Tasmania in 2016–2018, there is no information on IIH epidemiology [18]. Very little is known about the incidence of paediatric IIH. Among the two studies specifically assessing paediatric IIH, incidence rates were lower than seen among the general or adult populations (0.5–0.9/100,000 person-years) [19, 20].

Obesity and recent weight gain are well-established risk factors for IIH [21, 22], a condition that continues to increase globally [23]. However, IIH incidence in adults with obesity differs by sex [6], with much stronger correlations among females than males (77.8% of females with IIH meeting criteria for obesity vs 25.0% of males) [24]. Beyond mechanical effects of increased body mass, metabolic and hormonal factors associated with adipose tissue may also contribute to disease pathogenesis. Adipose-derived hormones such as leptin, which regulates appetite and energy balance, have been shown to be elevated in patients with IIH compared with BMI-matched controls [25, 26]. Leptin enters the cerebrospinal fluid via the choroid plexus and may influence CSF dynamics; impaired leptin signalling in states of raised intracranial pressure has been hypothesised to contribute to dysregulation of CSF production and absorption [27, 28]. In addition, the association between obesity and IIH incidence is considerably weaker in children, suggesting that the underlying pathophysiology may differ across age groups [29]. Hormonal changes associated with puberty may also influence IIH risk. In paediatric populations, increased adiposity and accelerated linear growth during early adolescence have been observed in patients with IIH, suggesting that metabolic and growth-related pathways may play a role in disease development [30]. Additionally, both gonadal hormones and androgen excess have been implicated in IIH, with associations reported with endogenous and exogenous exposure, pregnancy, polycystic ovarian syndrome and elevated androgen levels [31–35].

To remedy these gaps in the literature, we conducted a survey of the 14-year epidemiology of IIH among adult and paediatric populations in Victoria, Australia.

## Methods

### Participants

Victoria is the second most populous state of Australia, with an estimated population of 6.9 million in 2023. We conducted a retrospective study to examine the incidence of IIH among patients presenting to quaternary referral centres in Victoria, Australia, the Royal Victorian Eye and Ear Hospital (RVEEH) and the Royal Children’s Hospital (RCH). Both hospitals have state-based referral pathways. Data were collected between January 2010 and December 2023.

All newly diagnosed cases meeting the revised Friedman diagnostic criteria for definite or probable IIH were included [13]. This was defined by the presence of papilloedema on clinical examination; normal neuroimaging (no structural pathology or venous sinus thrombosis on MRI or CT venography) and an elevated lumbar puncture opening pressure (> 25 cm CSF) with normal cerebrospinal fluid (CSF) composition. Cases with an opening pressure < 25 cm CSF were included only if all other criteria for probable IIH were satisfied. Patients were excluded if they had evidence of secondary causes of raised intracranial pressure, including venous sinus thrombosis, structural intracranial pathology, or medication-related intracranial hypertension.

The study protocol was reviewed and approved by the Human Research Ethics and Research Committees of the Royal Victorian Eye and Ear Hospital (24/1608HL) and the Royal Children’s Hospital (MCC DERP Reference number 3689).

### Case identification and data collection

Cases were identified through triangulation of multiple sources to ensure comprehensive capture. First, emergency department (ED) presentations were screened by reviewing discharge summaries using ICD-10 code G93.2 (Benign Intracranial Hypertension (BIH) or Pseudotumor Cerebri) alongside relevant keywords including “pseudotumor cerebri,” “papilledema,” “benign intracranial hypertension,” “idiopathic intracranial hypertension,” and abbreviated terms “Idiopathic H” and “Idiopathic I.” Second, neuro-ophthalmology clinic records were systematically reviewed for specialist IIH referrals. Confirmatory investigations for each potential case were analysed, including MRI venography (MRV) or CT venography (CTV) to exclude secondary causes like venous sinus thrombosis; lumbar puncture results (opening pressures and CSF analysis) were extracted from pathology reports; and where available, optical coherence tomography (OCT) was used to objectively quantify papilledema severity.

### Estimation of annual incidence rates and statistical methods

Case numbers for each year from 2010 to 2023 were the numbers of IIH cases diagnosed that year at each of the two hospitals. The population numbers used to calculate crude and age-standardised rates were obtained from the Australian Bureau of Statistics (ABS) [36]. Incidence rates were calculated as the number of cases diagnosed each year divided by the corresponding mid-year population estimate for Victoria that year; these were calculated for adult- and paediatric-onset IIH, estimated per 100,000 person-years. Age-standardisation of incidence rates to the Australian 2021 standard population was done using the direct method [36]. Estimation of 95% CIs for all rates was done using the delta method [37]. Sex ratios (female: male) for crude and age-standardised incidence rates were also calculated.

Negative-binomial regression was used to model temporal trends in IIH incidence, accounting for mild overdispersion. The model showed a significant annual increase in incidence and a substantially lower incidence in males compared with females. Full model results and diagnostic assessments are provided in Supplementary Fig. 2.

Differences in incidence rates over time and between groups were assessed using negative-binomial regression, with the total population at risk (as measured or standard populations, as appropriate) included as an offset. Assessment of differences in incidence rate sex ratios over time was assessed by including a product term (*yearXsex*) in regression models, the significance of which denoted that of the change in sex ratio over time.

## Results

**Fig. 1:**
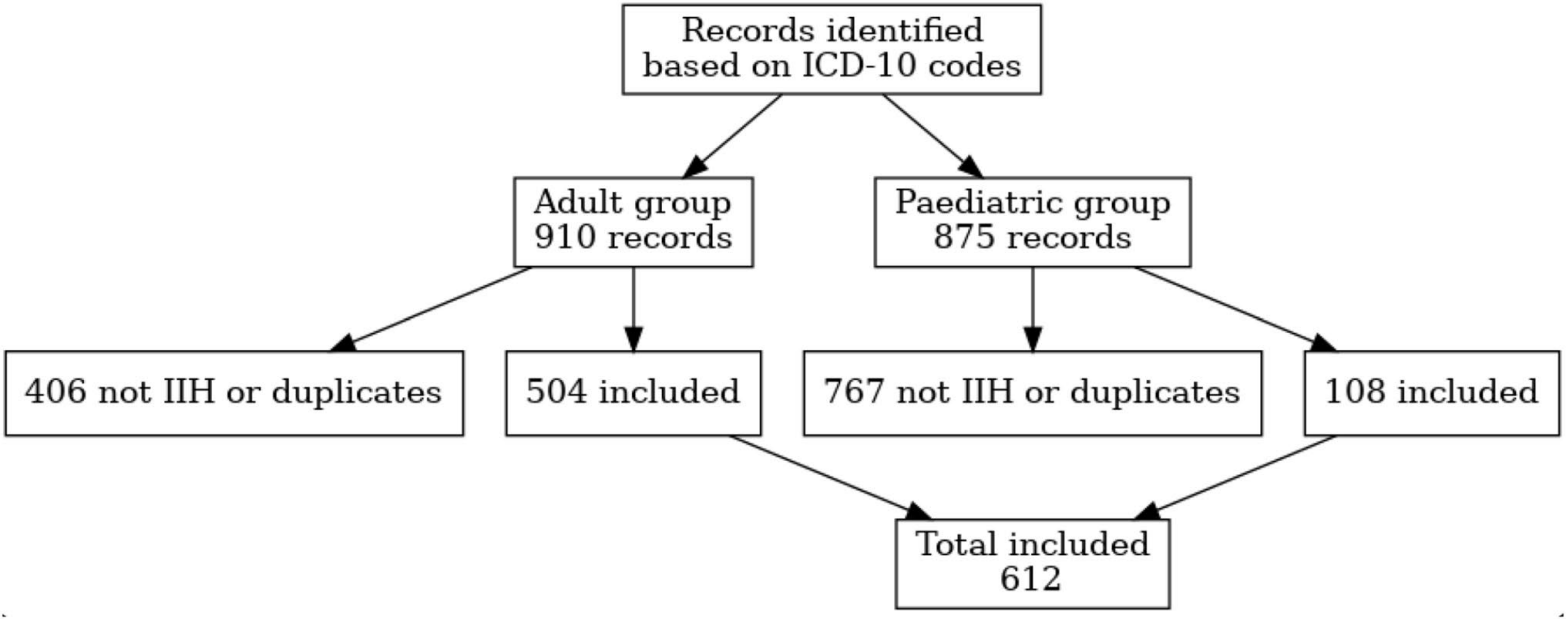
Flow diagram of case identification and inclusion. Records were identified using ICD-10 codes. After removal of non-IIH cases and duplicates, 504 adult and 108 paediatric cases were included, yielding a total cohort of 612 patients

A total of 910 adult records were initially identified using ICD-10 coding. After removal of 387 duplicate entries and exclusion of 523 records not meeting IIH diagnostic criteria or with alternate diagnoses, 504 valid IIH adult cases were included. In parallel, 875 paediatric records were screened, of which 751 were excluded (214 duplicate entries and 537 not meeting IIH diagnostic criteria). The latter were excluded due to alternative diagnoses, including pseudopapilloedema, intracranial tumours and other causes of optic disc swelling or intracranial pathology. This left 108 paediatric IIH cases eligible for inclusion. Together, they yielded a final study cohort of 612 patients (504 adults, 108 paediatric cases) (Fig. 1).

### Demographic and clinical characteristics

A total of 612 patients diagnosed with IIH were included in the study cohort. The demographics and clinical features are shown in Table 1. The majority were female (n = 548, 89.5%) and the mean age at diagnosis was 26.4 years (SD = 10.3), consistent with the recognised demographic predominance of IIH in females of reproductive age. The mean weight was 97.3 kg (SD = 28.6), and the mean body mass index (BMI) was 37 kg/m^2^ (SD = 8.6). 242 patients (39.5%) had a BMI > 30 kg/m^2^. The mean observed BMI in children, 31.9 (± 8.4), was lower than the BMI of the adult sample, 37.6 (± 8.4), with only 11 patients (9.8%) being obese. Pubertal status was not routinely documented in the medical records, which limited the ability to stratify paediatric patients according to pre- and post-pubertal status. For context, the paediatric cohort comprised 26 patients aged < 11 years, n = 50 aged 11–14, and *n* = 32 aged 14–18.

**Table 1:** Cohort demographics and characteristics

	Paediatric (< 18)	Adult (≥ 18)	All
Total number, *n*	108^†^	504	612
Sex			
Female, *n* (%)*	76 (70)	472 (94)	548 (90)
Male,* n* (%)	32 (30)	32 (6)	64 (10)
Age at diagnosis, years (mean ± SD)	12.4 (3.5)	28 (6.5)	26.3 (9.8)
Weight at diagnosis, kg (mean ± SD)	69.64 ± 27.4	104.17 ± 29.5	97.33 ± 28.6
BMI at diagnosis, kg/m^2^ (mean ± SD)	31.9 ± 8.4	37.6 ± 8.4	37.00 ± 8.6
Clinical phenotype			
Headache, *n* (%)	93 (86)	424 (84)	517 (84)
Blurred vision, *n* (%)	27 (25)	181 (36)	208 (34)
Transient visual obscurations, *n* (%)	12 (11)	137 (27)	149 (24)
Diplopia, *n* (%)	31 (29)	42 (8)	73 (12)
Cranial nerve VI palsy, *n* (%)	20 (19)	8 (2)	28 (5)
Pulsatile tinnitus, *n* (%)	13 (12)	172 (34)	185 (30)
Asymptomatic, *n* (%)	12 (11)	34 (7)	46 (8)
Clinical phenotype			
Headache, *n* (%)	93 (86)	424 (84)	517 (84)
Blurred vision, *n* (%)	27 (25)	181 (36)	208 (34)
Transient visual obscurations, *n* (%)	12 (11)	137 (27)	149 (24)
Diplopia, *n* (%)	31 (29)	42 (8)	73 (12)
Cranial nerve VI palsy, *n* (%)	20 (19)	8 (2)	28 (5)
Pulsatile tinnitus, *n* (%)	13 (12)	172 (34)	185 (30)
Asymptomatic, *n* (%)	12 (11)	34 (7)	46 (8)
Pregnant at diagnosis, *n* (%)	0	8 (1)	8 (1)
Diagnostic signs			
Frisen grade papilloedema, mean (range)	2.3 (1–5)	2.2 (1–5)	2.18 (1–5)
CSF opening pressure, cmCSF (mean ± SD)	37.4 ± 13.7	31.1 ± 8.6	32.23 ± 10
Visual assessment			
Visual acuity, LogMAR (mean ± SD)	0.05 ± 0.2	0.02 ± 0.2	0.03 ± 0.2
Visual field, PMD (mean ± SD)	− 5.21 ± 7	− 3.74 ± 5.0	− 3.91 ± 5.3
Optical coherence tomography retinal nerve fibre layer (OCT RNFL), µm (mean ± SD)	228 ± 120.7	180 ± 96.7	182 ± 97.6
Layer (OCT RNFL), µm (mean ± SD)			
Ganglion cell layer (GCL) thickness, µm (mean ± SD)	82 ± 14.7	77.81 ± 18.2	7.89 ± 18.1
Neuro-radiological signs^0^			
MRI findings	*n* = 93	*n* = 297	*n* = 390
Normal, *n* (%)	41 (44.1)	74 (24.9)	115 (29.5)
Optic nerve tortuosity, *n* (%)	8 (8.6)	37 (12.5)	45 (11.5)
Dilated optic nerve sheath, *n* (%)	45 (48.4)	148 (49.8)	193 (49.5)
Posterior scleral flattening, *n* (%)	22 (23.7)	73 (24.6)	95 (24.4)
Partially empty sella, *n* (%)	31 (33.3)	109 (36.7)	140 (35.9)
Transverse sinus stenosis, *n* (%)	45 (49.5)	149 (50.2)	194 (49.7)
Three MRI features of IIH	15 (16.1)	49 (16.5)	64 (16.4)
All MRI features			8 (2.05)
CT Brain/Venogram—Stenosis**	1 (1.1)	7 (2.4)	39 (23.4)
	*n* = 0 – 0	*n* = 165 – 39 (23.6)	

†Pubertal status was not routinely documented in the medical records. For context, the paediatric group comprised 26 patients who were aged < 11 years, 50 who were aged 11–14 years, and 32 who were aged 14–18

*Includes 3 Transgender males (sex assigned at birth—female). 2 of the 3 were on testosterone therapy at the time of diagnosis

0The neuro-radiological signs described in this study are based on the initial MRI reports obtained at the time of diagnosis

Only scans with the report available for review were included. (390 MRI’s + 167 CT’s)

**All paediatric patients underwent magnetic resonance imaging rather than computed tomography

### Treatment patterns

Medication to lower ICP was commenced at diagnosis in 100% of children, with 5% of those cases requiring dual treatment with acetazolamide and topiramate. In adults, 76% were commenced on treatment at diagnosis, with acetazolamide being the first-line drug in 69% (*n* = 345). Topiramate was started as the first-line treatment in 7.2% of adult cases (*n* = 36).

Surgical intervention was required in 12% of children (*n* = 13), with CSF shunt procedures (ventriculoperitoneal or lumboperitoneal (VP/LP) shunting) performed in nine patients (8%). Optic nerve sheath fenestration (ONSF) was performed in 3 children (2.7%), and transverse sinus stenting was performed in 1 case. In adults, surgical intervention was required in 30 cases (6%). ONSF was the first choice in 16 adults (3.2%) and VP/LP shunting in 13 adults (2.6%). Two patients required combined ONSF plus VP shunt (0.4%) and one patient had an urgent transverse sinus stent placed (0.2%). Across the 14-year study window, 43 surgical procedures were performed. The pre-pandemic decade (2010–2019) accounted for 22 procedures, averaging 2.2 per year. During the early pandemic period (2020–2021), 5 procedures were performed, yielding a comparable annual average of 2.5. However, surgical volume rose markedly in the later years, with 5 procedures in 2022 and 11 in 2023, indicating a substantial post-pandemic increase.

### Symptom patterns

The most common presenting symptom overall was headache, reported by 517 patients (84.5%), followed by blurred vision (n = 208, 34%), pulsatile tinnitus (*n* = 185, 30.2%), and transient visual obscurations (*n* = 149, 24.4%). Diplopia was observed in 42 adults (8%) with documented VIth nerve palsy in eight people (2%). However, these symptoms were more common in children with 31 (29%) reporting diplopia and 20 (19%) presenting with VIth nerve palsies. In the overall cohort, asymptomatic presentations occurred in 7.5% of patients, including 11% of children.

### Visual outcomes

Despite similar Frisen grade papilloedema (children = 2.3 versus adults = 2.2), RNFL thickness was substantially higher in children (228 µm (SD = 120.7)) compared to adults (180 µm (SD = 96.7)). Furthermore, paediatric patients had a higher mean absolute intracranial pressure (cmH2O) than adults (37.4 (SD = 13.7) versus 31.1 (SD = 8.6)). Children demonstrated greater visual field dysfunction in decibels (dB) with an average perimetric mean deviation (PMD) of − 5.2 (SD = 6.9) compared to adults with a PMD of − 3.74 (SD = 5.0). Visual acuity was preserved in both groups.

Across the full study period, structural and functional measures showed modest variation when examined by diagnostic era. Pre-pandemic values (2010–2019) demonstrated a mean MD of − 4.77 dB and mean RNFL thickness of 189.8 µm. During the early pandemic years (2020–2021), MD values were similar ( − 4.01 dB), while RNFL thickness was lower at 171.9 µm. In the subsequent years, MD remained broadly stable, measuring − 3.02 dB in 2022 and –3.44 dB in 2023, both comparable to the overall cohort average ( − 3.91 dB). RNFL thickness, however, showed a gradual increase from 177.7 µm in 2022 to 185.8 µm in 2023, approaching pre-pandemic levels and slightly exceeding the overall mean of 182 µm. Although these differences were modest, the higher RNFL values observed in 2023 may indicate that some patients presented with more pronounced swelling in the post-pandemic period, potentially reflecting delays in presentation or referral patterns.

### Incidence of IIH

**Fig. 2:**
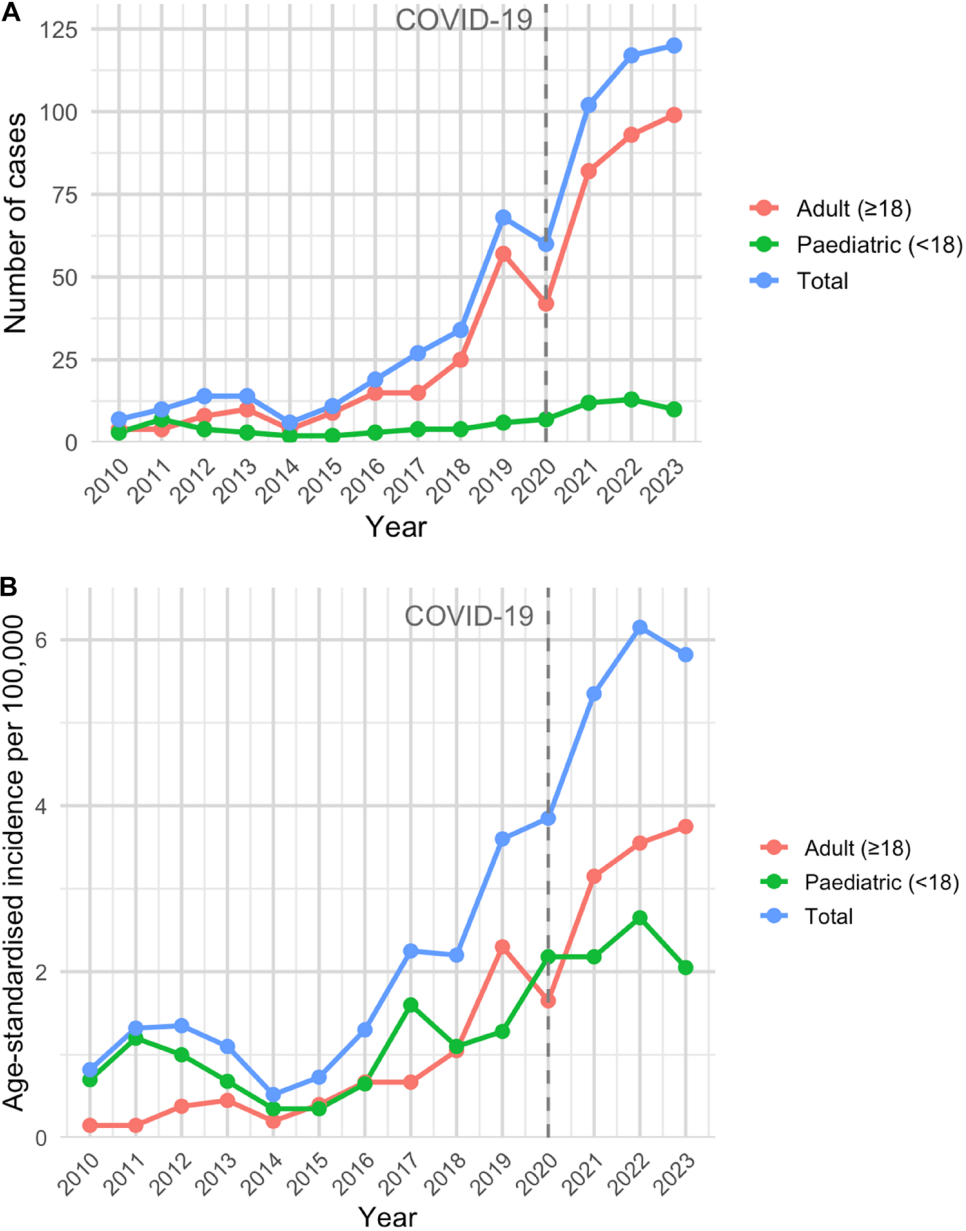
**a** Total case numbers by year and cohort. **b** Age-standardised incidence rates per 100,000 persons

Between 2010 and 2023, the age-standardised incidence rates increased sevenfold, from 0.86/100,000 in 2010–2014 to 6.53/100,000 in 2020–2023 (Fig. 2).

#### Incidence rates by sex

Incidence rates were consistently highest among females, with age-standardised incidence in adult females increasing almost 27-fold over the study period, from 0.14/100,000 in 2010 to 3.75/100,000 in 2023, compared to adult males among whom rates ranged 0.00–0.33/100,000. Among paediatric cases, incidence was higher in females than males throughout the study period, though the increase over time was roughly two-fold in both groups: females increased from 0.51 to 1.60/100,000 between 2010 and 2023, whereas males increased from 0.16 to 0.46/100,000.

In sex-stratified analyses, IIH incidence increased significantly over time in both females and males (*p* < 0.001). However, while absolute incidence rates were higher among females, there was no evidence that the rates of increase in incidence rates differed by sex (p_difference_ = 0.89).

Consistent with this pattern, the adult-to-paediatric incidence rate ratio among females increased from approximately 1:1 in 2010 to around 10:1 by 2022–2023, reflecting a disproportionate rise in adult female incidence relative to paediatric female.

The highest rates were seen among females aged 18–39 years, in whom incidence rose from 0.11/100,000 (95%CI 0.00–0.59) in 2010 to a peak of 7.49/100,000 (95%CI 5.91–9.35) in 2022, before declining slightly to 6.79/100,000 (95%CI 5.34–8.52) in 2023.

Overall, both absolute incidence rates and the magnitude of increase over time were greatest among adult females, indicating a pronounced and sex-specific shift in IIH burden towards younger adult females.

#### IIH incidence rates, by age groups

Although IIH incidence rates increased during the 2010–2023 period among both adult and paediatric populations, the magnitude of increase was greater among adults than among paediatric populations (p_difference_ < 0.001). Among adults, incidence rates increased 32-fold between 2010 and 2023 (p_trend_ < 0.001), from 0.07/100,000 (95%CI 0.02–0.21) to 2.30/100,000 (95%CI 2.05–2.75). Paediatric incidence rates increased threefold (p_trend_ < 0.001) from 0.33/100,000 (95%CI 0.10–1.21) in 2010 to a peak of 1.33/100,000 in 2022 (95%CI 0.81–2.19) and 1.01/100,000 in 2023 (95%CI 0.58–1.70).

**Fig. 3:**
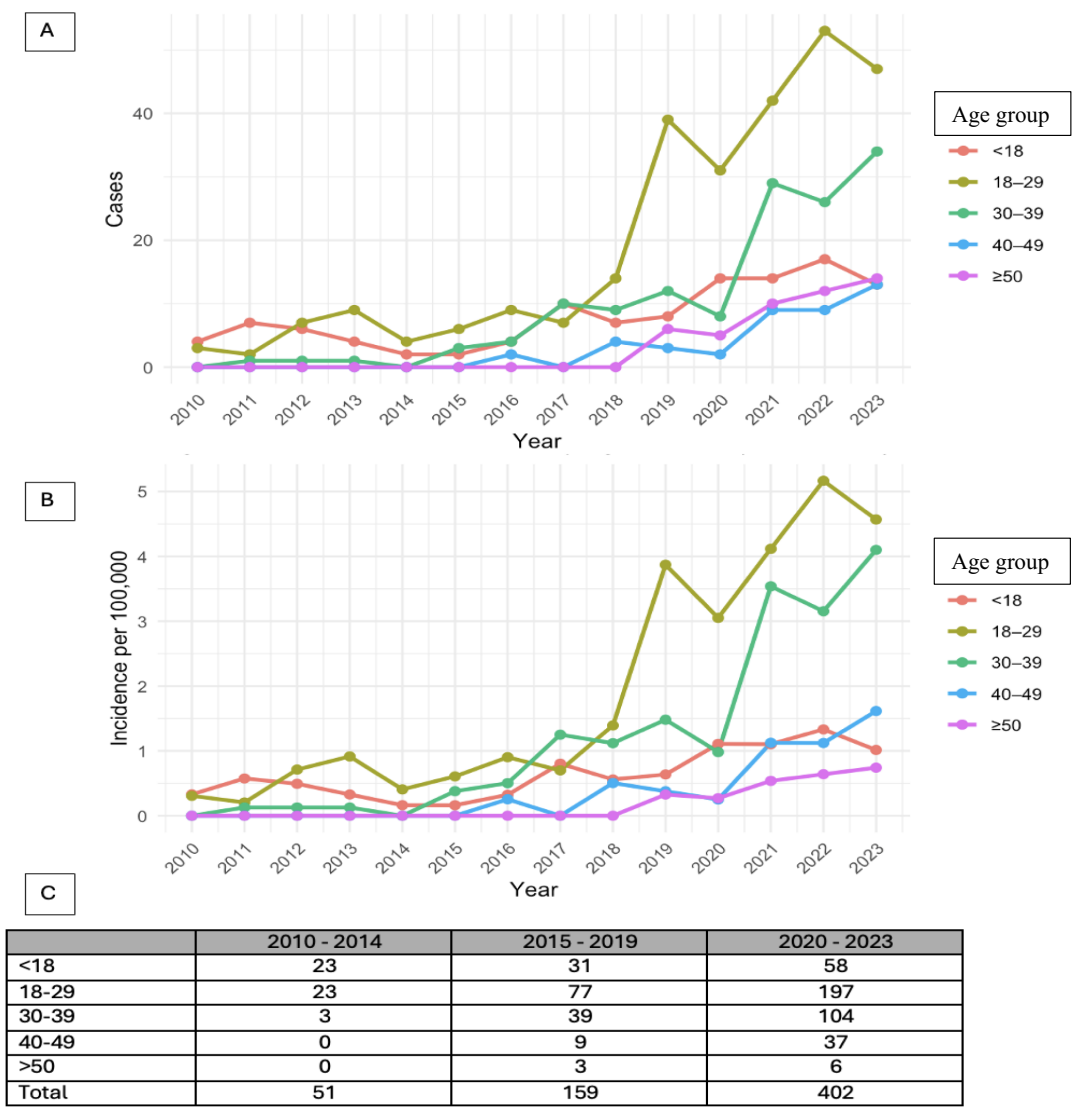
Annual IIH incidence by age group, 2010–2023: (**A**) incident case numbers; (**B**) incidence rates/100,000 persons; and (**C**) incident case numbers across three periods (2010–2014, 2015–2019, 2020–2023)

Further interrogating the age-specific effects, Fig. 3 shows the distribution of IIH incidence with adult populations separated into roughly 10-year age groups, compared to those under 18 years old. From 2010 to 2017, numbers of incident cases (Fig. 3a) and age-standardised incidence rates (Fig. 3b) were statistically identical between age groups. However, a marked increase became apparent from 2018, particularly among young adults aged 18–29 (increasing 1.1 to 4.26 in 2023), and from 2020 onwards for other adult age groups, especially those aged 30–39 (increasing 0.98 to 4.10 in 2023. Although incidence was consistently higher in females than males across all age groups, the rate of increase over time did not differ significantly by sex (sex-by-year interaction *p* = 0.891) (Table 2).

**Table 2:** Annual IIH incidence for 2010–2023, adult vs paediatric cases, overall and by sex

Group	2010	2011	2012	2013	2014	2015	2016	2017	2018	2019	2020	2021	2022	2023
Female adult (≥ 18)														
Case numbers	3	3	8	10	4	9	15	15	25	57	42	82	93	99
Crude incidence rate (/100,000 persons) (95% CI)	0.14 (0.03–0.40)	0.14 (0.03–0.40)	0.36 (0.16–0.72)	0.45 (0.22–0.84)	0.18 (0.05–0.46)	0.40 (0.19–0.77)	0.67 (0.38–1.11)	0.67 (0.37–1.10)	1.11 (0.72–1.63)	2.51 (1.90–3.25)	1.84 (1.32–2.48)	3.57 (2.84–4.43)	4.02 (3.25–4.93)	4.26 (3.46–5.19)
Age–standardised incidence rate (/100,000 persons) (95% CI)	0.14 (0.03–0.41)	0.14 (0.03–0.41)	0.37 (0.16–0.72)	0.45 (0.22–0.83)	0.18 (0.05–0.47)	0.40 (0.18–0.77)	0.65 (0.36–1.07)	0.65 (0.36–1.07)	1.06 (0.69–1.57)	2.30 (1.74–2.98(	1.65 (1.19–2.23)	3.15 (2.50–3.91)	3.53 (2.85–4.33)	3.75 (3.04–4.58)
Female paediatric (< 18)														
Case numbers	3	7	4	3	2	2	3	4	4	6	7	12	13	10
Crude incidence rate (/100,000 persons) (95% CI)	0.51 (0.10–1.48)	1.18 (0.47–2.43)	0.67 (0.18–1.72)	0.50 0.10–1.47)	0.33 (0.04–1.21)	0.33 (0.04–1.20)	0.50 (0.10–1.45)	0.66 (0.18–1.69)	0.66 (0.18–1.68)	0.98 (0.36–2.13)	1.14 (0.46–2.34)	1.94 (1.00–3.39)	2.09 (1.11–3.58)	1.60 (0.77–2.95)
Age–standardised incidence rate (/100,000 persons) (95% CI)	0.51 (0.11–1.48)	1.18 (0.47–2.43)	0.67 (0.18–1.72)	0.50 (0.10–1.45)	0.33 (0.04–1.19)	0.33 (0.04–1.18)	0.50 (0.10–1.45)	0.66 (0.18–1.69)	0.66 (0.18–1.68)	0.98 (0.36–2.14)	1.14 (0.46–2.36)	1.89 (0.98–3.30)	2.04 (1.09–3.49)	1.60 (0.77–2.94)
Male adult (≥ 18)														
Case numbers	0	0	0	0	0	0	0	2	2	3	4	6	7	8
Crude incidence rate (/100,000 persons) (95% CI)	0.00 (0.00–0.18)	0.00 (0.00–0.18)	0.00 (0.00–0.18)	0.00 (0.00–0.17)	0.00 (0.00–0.17)	0.00 (0.00–0.17)	0.00 (0.00–0.17)	0.09 (0.00–0.33)	0.09 (0.00–0.33)	0.14 (0.03–0.40)	0.18 (0.05–0.47)	0.27 (0.10–0.59)	0.32 (0.13–0.65)	0.36 (0.16–0.71)
Age–standardised incidence rate (/100,000 persons) (95% CI)	0.00 (0.00–0.00)	0.00 (0.00–0.00)	0.00 (0.00–0.00)	0.00 (0.00–0.00)	0.00 (0.00–0.00)	0.00 (0.00–0.00)	0.00 (0.00–0.00)	0.09 (0.01–0.32)	0.09 (0.01–0.31)	0.13 (0.03–0.38)	0.17 (0.05–0.44)	0.25 (0.09–0.55)	0.29 (0.12–0.61)	0.33 (0.14–0.65)
Male paediatric (< 18)														
Case numbers	1	0	2	1	0	0	1	6	3	2	7	2	4	3
Crude incidence rate (/100,000 persons) (95% ci)	0.16 (0.00–0.89)	0.00 (0.00–0.59)	0.32 (0.04–1.15)	0.16 (0.00–0.89)	0.00 (0.00–0.59)	0.00 (0.00–0.58)	0.16 (0.00–0.88)	0.94 (0.34–2.04)	0.47 (0.10–1.36)	0.31 (0.04–1.12)	1.08 (0.43–2.22)	0.31 (0.04–1.11)	0.61 (0.17–1.56)	0.46 (0.09–1.33)
Age–standardised incidence rate (/100,000 persons) (95% CI)	0.16 (0.00–0.90)	0.00 (0.00–0.00)	0.32 (0.04–1.16)	0.16 (0.00–0.88)	0.00 (0.00–0.00)	0.00 (0.00–0.00)	0.16 (0.00–0.87)	0.93 (0.34–2.03)	0.46 (0.10–1.35)	0.30 (0.04–1.08)	1.05 (0.42–2.16)	0.30 (0.04–1.07)	0.60 (0.16–1.54)	0.46 (0.10–1.34)
All persons case numbers	7	10	14	14	6	11	19	27	34	68	60	102	117	120
Total crude incidence rate (/100,000 persons) (95% CI)	0.13 (0.05–0.26)	0.18 (0.09–0.33)	0.25 (0.14–0.43)	0.25 (0.14–0.43)	0.11 (0.04–0.23)	0.20 (0.10–0.35)	0.34 (0.20–0.53)	0.48 (0.31–0.70)	0.60 (0.41–0.84)	1.19 (0.92–1.51)	1.04 (0.80–1.34)	1.77 (1.44–2.14)	2.02 (1.67–2.42)	2.06 (1.71–2.46)
Total age–standardised incidence rate (/100,000 persons) (95% CI)	0.13 (0.03–0.22)	0.18 (0.07–0.29)	0.25 (0.12–0.38)	0.25 (0.12 –0.38	0.11 (0.02–0.19)	0.20 (0.08–0.31)	0.34 (0.19–0.49)	0.48 (0.30–0.66)	0.60 (0.40–0.80)	1.19 (0.91–1.48)	1.04 (0.78–1.31)	1.80 (1.46–2.15)	2.02 (1.65–2.38)	2.08 (1.71–2.45)

## Discussion

This study provides the first comprehensive population-level assessment of IIH incidence across both adult and paediatric populations over 14 years in Victoria, Australia, and is also the first study assessing IIH epidemiology before, during and after the COVID-19 pandemic. Overall, we observed a striking and sustained rise in IIH incidence, driven predominantly by young adult females, with comparatively smaller changes in other demographic groups. The rise in incidence was sustained rather than transient and remained elevated in the later years of the study period, suggesting a shift toward a higher underlying disease burden. Together, these findings indicate that IIH incidence in Victoria has increased substantially over the past decade while retaining the characteristic demographic profile described in previous literature [3, 4].

Our results indicate disproportionately rapid growth in young adult females, far exceeding changes in other demographic groups. The greatest incidence increase was observed among females aged 18–39 years, among whom age-standardised incidence rates rose from 0.11/100,000 in 2010 to a peak of 7.49/100,000 in 2022, before a modest decline to 6.79/100,000 in 2023. Importantly, the marked rise in incidence was not explained by shifts in the demographic structure of the Victorian population, as age-standardised incidence rates showed similar dynamics. In particular, standardised incidence rates increased almost seven-fold among females (0.86/100,000 in 2010–2014 to 6.53/100,000 in 2020–2023) while incidence rates among adult males never exceeded 0.36/100,000. This aligns with the known epidemiology of IIH [12, 15], wherein females have long comprised the majority of cases, and which persists here despite the overall expansion in case numbers [3]. These observations suggest an intensification of an established demographic distribution rather than a shift in disease phenotype or risk profile. Notably, incidence in this group has remained elevated since 2021, suggesting a transition to a new, higher steady state rather than a transient fluctuation.

Paediatric IIH incidence rates rose more modestly (peaking at 1.33/100,000 in 2022) and remained lower than in adults. In addition, the female predominance was lower in children—71% compared to 94% in adults. This may suggest that sex-related risk factors become more influential after puberty and this needs to be examined further in future studies. Paediatric IIH presented with higher rates of focal neurological signs such as diplopia (29% vs 8%) and cranial nerve VI palsy (18% vs 2%). Therapeutically, this translated to an observed increase in the use of acetazolamide in paediatric cases. These distinctions support the concept of paediatric IIH as a distinct phenotype with specific diagnostic and management considerations [19, 38].

Sex-specific analyses emphasised that the contemporary rise in IIH burden is almost entirely driven by female adults, with the female-to-paediatric incidence ratio increasing from approximately 1:1 in 2010 to around 10:1 by 2022–2023. Male incidence rates remained low and variable due to small case numbers. These sex- and age-specific patterns are consistent with the characteristic epidemiological profile of IIH and align with the literature identifying reproductive-aged females as the group most affected [3, 4].

The drivers of the increased incidence of IIH are multifactorial. Firstly, the prevalence of obesity continues to increase. In Australia, the proportion of adults who are overweight or obese has increased (Australian Bureau of Statistics (ABS); Supplementary Fig. 1), from approximately 61% to 65–67% over the past decade, with similar upward trends observed in children, in whom prevalence has risen from approximately 20% in 1995 to 28% in 2022 [39, 40]. These patterns mirror global trends, with projections suggesting that by 2030 up to 50% of adults and a substantial proportion of children and adolescents will be affected by overweight or obesity. While this supports obesity as a key driver, the magnitude and rapidity of the increase in IIH incidence exceed the more gradual rise in obesity rates, suggesting additional factors, such as increased disease recognition, diagnostic intensity, including greater access to neuro-ophthalmology services and advanced imaging, and broader metabolic changes, are also contributing. Furthermore, BMI may underestimate risk, with emerging evidence implicating visceral adiposity and inflammatory pathways in IIH pathophysiology.

We noted a further steep increase in incidence from 2020 onwards that aligns with the onset of the COVID-19 pandemic. Victoria experienced some of the most prolonged and stringent COVID-19 lockdowns worldwide, a factor which may have influenced patterns of weight gain and healthcare access during the study period, including via impacts on exercise, diet, and mental health. Indeed, the COVID-19 pandemic was associated with accelerated weight gain, with one study in South Korea showing 11% of adults transitioning into overweight or obesity and 42.5% of those with pre-existing overweight or obesity reporting further weight gain [41]. This increase was linked to pandemic-related behavioural changes, including higher stress, increased social isolation, and greater use of online food delivery and leisure platforms. These findings have importance in the context of the childhood obesity epidemic [42]. Early-onset obesity that leads to paediatric IIH can result in severe complications such as vision loss and chronic headaches. However, weight gain alone is unlikely to fully explain the observed increase in IIH. Emerging evidence suggests that COVID-19 infection itself may be associated with the development or worsening of IIH, even in the absence of traditional risk factors like weight gain and vitamin A exposure. Case reports and small series have described new-onset or worsening IIH in COVID-19 positive patients, including individuals who had experienced recent significant weight loss. Although the underlying mechanism remains incompletely understood, proposed pathways include tropism of SARS-CoV-2 for choroid plexus and CSF-brain barrier tissues, with resultant dysfunction potentially disrupting CSF homeostasis [43]).

In our cohort, a total of 43 surgical procedures were performed between 2010 and 2023, with notable variation across the pre-pandemic, pandemic, and post-pandemic periods. Surgical activity remained relatively stable during the early pandemic years, with five procedures performed across 2020–2021, comparable to the pre-pandemic average of 2.2 procedures per year. This contrasts with reports from several international centres describing substantial reductions in elective and semi-urgent neuro-ophthalmic and neurosurgical activity during the initial phases of the COVID-19 pandemic, largely attributed to service reallocation, reduced operating room capacity, and patient hesitancy to seek care. In our dataset, however, the most pronounced changes occurred after the acute pandemic period, with procedure numbers increasing to 5 in 2022 and rising further to 11 in 2023. This represents the highest annual volume observed. This post-pandemic increase aligns with global observations of a rebound in surgical activity as health systems addressed accumulated backlogs and patients who had deferred care re-entered the system. A UK study reported a 367% increase in emergency cerebrospinal fluid shunting procedures following lockdown, alongside worsening papilloedema, weight gain and adverse mental health outcomes [44]. The pattern in our cohort therefore suggests that, while early pandemic restrictions did not markedly reduce surgical intervention rates, the subsequent years were characterised by a substantial rise in surgical volume, likely reflecting the larger case numbers, but it may also be related to delayed presentations, increased disease severity at the time of referral, and restoration of full surgical capacity. While causality cannot be inferred, this temporal clustering raises concerns for increased disease burden and the downstream impact of healthcare disruption.

Our findings are broadly consistent with previous international studies. A meta-analysis by Chen and Wall reported incidence rates ranging from 0.03 to 2.2/100,000 person-years from studies conducted over 1984–2011 [16]. One other Australian IIH incidence study from southern Tasmania reported an average 2016–2018 annual incidence rate of 5.4/100,000 persons per year [18]. This incidence rate is markedly higher than our or others’ IIH incidence studies, and may be attributable to differences in case ascertainment, or potentially regional variation in risk factors such as obesity.

## Strengths and limitations

The strengths of our study include the large sample size, statewide referral base, and triangulation of multiple data sources to maximise case capture. This dataset also represents the most comprehensive overview of IIH incidence in Victoria, and indeed Australia, to date. Furthermore, it is the first study to include a pandemic population. However, limitations inherent to retrospective designs remain. Under-ascertainment is possible, particularly for milder cases managed outside Victorian quaternary centres, or outside Victoria altogether and pandemic-related disruptions to healthcare access may have reduced case detection or delayed presentation during the study period. Future work could further refine national estimates through capture–recapture methodology, which allows quantification of the degree of under-ascertainment by comparing overlapping cases across multiple independent data sources (e.g. hospital records, imaging databases, lumbar puncture logs). Alternatively, a prospective, multicentre surveillance study spanning several major Australian cities (e.g. Melbourne, Sydney, Brisbane) or a national registry-based approach would enable more complete ascertainment and allow direct comparison across jurisdictions.

Nevertheless, several methodological considerations should be acknowledged. A key strength of this study is the availability of BMI data at diagnosis, allowing clearer characterisation of anthropometric patterns in IIH. However, BMI was not reliably recorded at subsequent visits, limiting our ability to assess weight trajectories over time. Additionally, more detailed metabolic markers (e.g. lipid profiles or glycaemic indices) were not consistently available, restricting exploration of potential mechanisms. Future prospective studies with systematic follow-up measurements would help address these limitations.

Over the 14-year observation period of this study, optical coherence tomography (OCT) technology underwent significant advances, meaning that earlier scans may not be directly comparable in accuracy or resolution to more recent measurements. Furthermore, the COVID-19 pandemic and associated lockdowns are likely to have influenced patterns of presentation and diagnosis, with some cases potentially being delayed into subsequent years. Although negative binomial regression was used to evaluate differences in incidence over time and between sexes, these analyses relied on aggregate census-derived population denominators, which limit the granularity with which population dynamics within age and sex strata can be examined. As such, findings should be interpreted as population-level associations rather than evidence of causality, consistent with ecological study design.

## Conclusion

In conclusion, this study demonstrates a substantial and sustained rise in the incidence of IIH in both adults and children in an Australian population over 14 years of follow-up, with the greatest increase observed among young adult females. These epidemiological changes have important implications for health service planning, including the need for adequate neuro-ophthalmology and neurology capacity, timely access to visual field testing and OCT, and provision for surgical management in selected cases.

Although the proportion of patients who develop significant visual impairment is low and typically associated with severe disease, delays in access to care may increase this risk. In our cohort, surgical intervention rates were higher in the post-pandemic years compared with earlier periods. While this may reflect delayed presentation in some patients, it may also simply correspond to the increased number of cases seen during this time, and therefore should be interpreted cautiously. Continued surveillance and targeted public health strategies addressing modifiable risk factors, particularly obesity, will be essential to support and manage the growing clinical burden of IIH.

## Key messages

### What is already known on this topic

Idiopathic intracranial hypertension (IIH) incidence is increasing in northern hemisphere studies. IIH incidence rates are known to correlate with obesity rates.

### What this study adds

This study provides the first population-based estimates of the incidence rates of IIH in Victoria, Australia over a 14-year period that includes the COVID-pandemic. The annual incidence rates for IIH rose sevenfold, from 0.86/100,000 person-years in 2010–2014 to 6.53/100,000 in 2020–2023. The greatest increases in IIH incidence were observed among females aged 18–39 years.

This work particularly adds to the limited literature on childhood-onset IIH. Childhood cases increased by threefold from 0.33/100,000 in 2010 to 1.33/100,000 in 2020 and 1.01/100,000 in 2023. Children with IIH were more likely than adults to present with focal neurological signs such as sixth nerve palsy.

### How this study might affect research, practice, or policy

Our results provide the first estimates of the incidence of IIH in a first-world Southern Hemisphere population. Childhood-onset IIH is distinct from adult IIH, providing valuable pathophysiological insights. Critical research is needed to understand the pathophysiology of IIH overall and within key population subgroups to counteract this rapidly rising neurological disease.

## Supplementary Information

Below is the link to the electronic supplementary material.

Supplementary file1 (DOCX 96 KB)

## Data Availability

The datasets generated and/or analysed during the current study are not publicly available due to ethical and privacy restrictions related to patient confidentiality, but are available from the corresponding author on reasonable request and subject to institutional and ethics approval.
