# Supplementary material for: Incidence of idiopathic intracranial hypertension (IIH) in adults and children: a 14-year population-based study
Source: J Neurol. 2026 Jun 13;273(7):394. doi: 10.1007/s00415-026-13901-2 (PMC13264580; doi:10.1007/s00415-026-13901-2)

**Supplementary data**

**Figure 1:** Proportion of Adults Obese or Overweight over time

Supplementary appendix 1. Negative-binomial regression model.

We fitted a negative-binomial regression model to account for mild overdispersion (deviance/df=1.33) in the annual incidence of IIH, using case counts stratified by year and sex, with the at-risk population (aged 15–50 years) included as an offset. The model demonstrated a statistically significant increase in IIH incidence over time, with an estimated annual incidence rate ratio (IRR) of 1.27 (95% CI: 1.22–1.31, p<0.001), indicating a 27% increase in incidence per year. Males had a significantly lower incidence compared with females, with an IRR of 0.12 (95% CI: 0.085–0.159, p<0.001), corresponding to an ~88% lower rate. The model showed good fit (residual deviance=33.2 on 25 df), and the estimated dispersion parameter (θ=28.99, SE=22.58) suggested only mild overdispersion. Model adequacy was also assessed by inspection of Pearson residuals, which were symmetrically distributed around zero without temporal structure (**Figure** **2**). These findings were consistent with results from the quasi-Poisson model, which produced nearly identical incidence rate ratios for both year (IRR≈1.27, p<0.001) and sex (IRR≈0.12, p<0.001), supporting the robustness of the observed trends and justifying the use of a negative-binomial model over a standard Poisson regression.

**Figure 2**: Pearson residuals from the negative-binomial regression model (B). Predicted values closely tracked observed counts, and residuals were centred around zero without systematic temporal trends, supporting model adequacy (Pearson χ²/df=1.22).


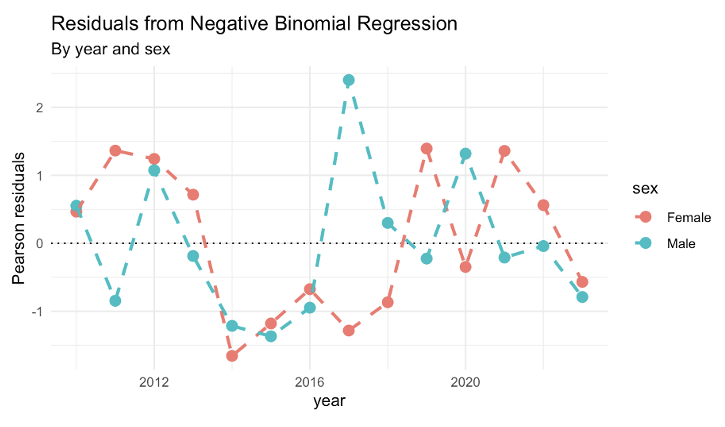

Supplement: Supplementary file 1 — Supplementary file1 (DOCX 96 KB) [file 415_2026_13901_MOESM1_ESM.docx]
